# Anatomical and morphological spine variation in Gymnocalycium kieslingii subsp. castaneum (Cactaceae)

**DOI:** 10.3897/phytokeys.69.8847

**Published:** 2016-08-18

**Authors:** Roman Gebauer, Radomír Řepka, Radek Šmudla, Miroslava Mamoňová, Jaroslav Ďurkovič

**Affiliations:** 1 Department of Forest Botany, Dendrology and Geobiocoenology, Mendel University, Zemědělská 3, 61300 Brno, Czech Republic; 2 Department of Wood Science, Technical University, 96053 Zvolen, Slovak Republic; 3 Department of Phytology, Technical University, 96053 Zvolen, Slovak Republic

**Keywords:** Areole, fiber, minimum sample size, spine cross-section, spine morphogenesis, spine surface

## Abstract

Although spine variation within cacti species or populations is assumed to be large, the minimum sample size of different spine anatomical and morphological traits required for species description is less studied. There are studies where only 2 spines were used for taxonomical comparison amnog species. Therefore, the spine structure variation within areoles and individuals of one population of Gymnocalycium kieslingii subsp. castaneum (Ferrari) Slaba was analyzed. Fifteen plants were selected and from each plant one areole from the basal, middle and upper part of the plant body was sampled. A scanning electron microscopy was used for spine surface description and a light microscopy for measurements of spine width, thickness, cross-section area, fiber diameter and fiber cell wall thickness. The spine surface was more visible and damaged less in the upper part of the plant body than in the basal part. Large spine and fiber differences were found between upper and lower parts of the plant body, but also within single areoles. In general, the examined traits in the upper part had by 8–17% higher values than in the lower parts. The variation of spine and fiber traits within areoles was lower than the differences between individuals. The minimum sample size was largely influenced by the studied spine and fiber traits, ranging from 1 to 70 spines. The results provide pioneer information useful in spine sample collection in the field for taxonomical, biomechanical and structural studies. Nevertheless, similar studies should be carried out for other cacti species to make generalizations. The large spine and fiber variation within areoles observed in our study indicates a very complex spine morphogenesis.

## Introduction

Spines may be considered one of the most characteristic morphological structures of the *Cactaceae* family. Cactus spines are the modified bud scales of an axillary bud, originating from primordia which are morphologically indistinguishable from the leaf primordia (Mauseth 2006). Spines contain just two cell types, which never occur in long-shoot leaves of cacti: libriform fibers and sclerified epidermis cells (Mauseth 2006). The cactus spine epidermis lacks stomata. In a few species, some spine epidermis cells elongate outward as trichomes (Sotomayor and Arredondo 2004, Mauseth 2006, Řepka and Gebauer 2012). The epidermis can be continuous, divided into single cell elements or transversely fissured, and such fissures extend deeply into the underlying sclerenchyma (Barthlott 1979).

Spines are not only a lifeless part of the plant body but have several important functions. They provide defense against herbivores (Norman and Martin 1986, Gibson and Nobel 1986), protect the sensitive meristems from freezing temperatures (Loik and Nobel 1993, Mauseth 2006) and shade the plants to avoid temperature stress (Gibson and Nobel 1986, Mauseth 2006). Consequently, each cactus must evolve its spine coverage pattern in order to maximize its photosynthetic efficiency within its own habitat (Menezes et al. 2015). The spine surface of *Opuntia* is also constructed as an efficient system to collect fog and to drive water droplets towards the spine base, where they are absorbed (Ju et al. 2012). Moreover, the areole position on the plant body may reveal past physiological and climatic variation since new spines develop on the top of the cactus body whereas the oldest spines are situated in the basal part (English et al. 2007). The exception are tree-like species of *Opuntia* and *Quiabentia*, which continue producing spines from their lower areoles even in old age (Rowley 2003).

The spine diversity within the family is truly spectacular and spine anatomical and morphological traits are useful tools for taxonomists (e.g. Hunt et al. 2006, Mosco 2009, Řepka and Gebauer 2012). Although, there is evidence that spine development is influenced by genetic (Mihalte and Sestras 2012) and environmental conditions as water availability or solar radiation can modified spine growth (e.g. Peharec et al. 2010, Menezes et al. 2015). Nevertheless, to the best of our knowledge, there is no prior study analyzing spine and fiber structure variation within areoles, individuals or populations, with the exception of variation in spine number and length (e.g. Schmalzel et al. 2004, Hunt et al. 2006, Baker and Butterworth 2013, Menezes et al. 2015). Although the variation in spine anatomical and morphological traits within a population or species is assumed to be large (Mauseth 2006), there are studies where only 2 spines were used for *Turbinicarpus* species comparison (Mosco 2009) or for the assessment of spine stiffness (Schlegel 2009).

Spine and fiber variation within areoles and individuals was studied in one Gymnocalycium kieslingii subsp. castaneum (Ferrari) Slaba population with the intent to solve three questions: (1) does areole position on the plant body play an important role in spine and fiber variation?; (2) are spine and fiber traits less variable within an areole than between areoles and individuals?; (3) how many spines need to be collected for an analysis of specific traits? Our results will provide useful information for spine sample collection in the field for taxonomical, biomechanical, physiological or structural studies.

## Methods

### Plant material

A single representative of the nominate subgenus of *Gymnocalycium*, Gymnocalycium kieslingii subsp. castaneum was chosen for the study. This is an endemic taxon of the Argentinian province of La Rioja, which is taxonomically clear with relatively low morphological variability. It grows in 13 populations occupying fairly narrow ecological niche. It grows on the poor and highly permeable sandy soils without humus, blended with skeleton basement rock, generated by the disintegration of granitoids on the slopes of the Sierra de Velasco Mts. The climate is semi-arid and the mean annual precipitation is 360 mm. The mean annual temperature is 20 °C with 2,800 hours of sunshine (http://www.arquinstal.com.ar/atlas/datos/larioja.html). Plants are uniformly found under/or outsides of *Larrea cuneifolia* Cav. in the phytogeographical district Monte (Cabrera 1976) at altitudes of 1250–1550 m. Distribution area is relatively small (cca 760 km^2^) compared to other species of the subgenus. It lies between city of Aimogasta in the north and city of Villa Sanagasta in the south.

The plant usually forms flattened spherical bodies 60–90 mm in diameter. It rarely achieves a greater height than width (Till 1990). The plant material was collected from one population at the sandy depression of Bolsón de Huaco, SSW of the village of El Huaco in the northern part of the La Rioja Province, at altitude of 1260 m (67°07’S; 29°22’W). Fifteen plants were randomly selected on a ca. 700 m line along the road (north-south), at distances of 50 m from each other. From each plant, one areole from the basal, middle and upper part on the northern face of the plant body were sampled (i.e. 45 areoles in total). Additional areoles were sampled from 5 plants at each examined position for scanning electron microscopy (i.e. 15 areoles). Only fully developed areoles were collected.

### Scanning electron microscopy (SEM)

The whole areole was mounted on specimen stubs, sputter-coated with gold, and observed with high-vacuum SEM using a VEGA TS 5130 instrument (Tescan, Czech Republic) operating at 15 kV. Images of the whole areole and detailed images of the spine surface in the middle and top spine parts were made. From these images, epidermis characteristics, shape of epidermis cells, presence and type of trichomes and presence of fissures were determined.

### Light microscopy

**Figure 1.**
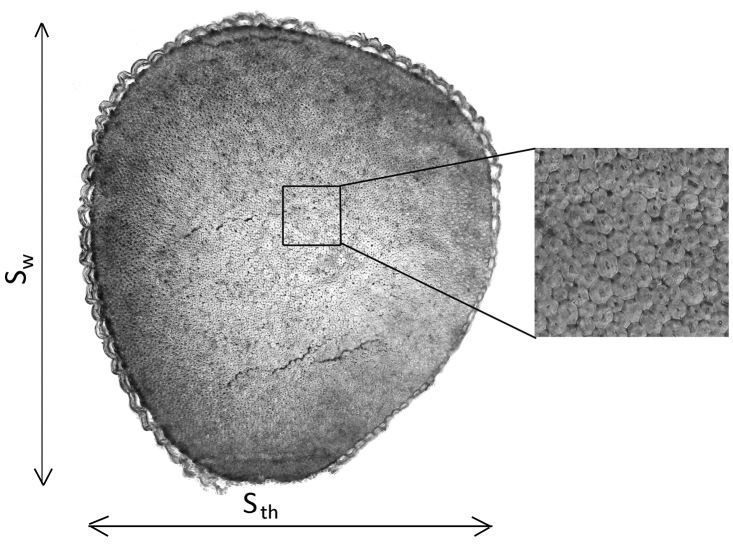
Measured spine traits: spine width (S_w_) and spine thickness (S_th_). Fibers are shown in the detailed picture.

A 5% solution of hydrochloric acid was used to soften spines before sectioning. In this solution, two days of soaking was sufficient to soften the spines for anatomical analysis. The spine length (S_l_) was measured before making cross-sections. Handmade cross-sections were taken from the spine base and were examined under a bright field microscope (Olympus BX51, Olympus Czech Group, Czech Republic) at magnifications up to 400× and were photographed using a digital camera (Olympus E-330, Olympus Czech Group, Czech Republic) connected to a computer with the QuickPhotomicro 2.3 software (Promicra, Czech Republic). Spine width (S_w_), spine thickness (S_th_), spine cross-section area (S_a_), spine circumference (S_c_), fiber maximum and minimum diameter (F_max_ and F_min_), and cell wall thickness (CW_th_) were measured using the ImageTool 3.00 software (UTHSCSA, USA) (Fig. 1). Ten fibers were measured in each spine. Only the largest fibers were measured as fibers at the end taper. Spine and fiber roundness (S_r_ and F_r_, respectively) were defined as the S_w_/S_th_ and F_max_/F_min_ ratio, respectively, with a ratio of 1 denoting a perfectly round cross-section and larger ratios indicating a more ellipsoidal shape. In total, 245 spines were examined.

### Statistical analysis

The first step was the calculation of fundamental descriptive characteristics using linear mixed effect models (LME). In these models, all of the traits come from a nested design; therefore, we used LME to avoid the problem of pseudoreplication (Hurlbert 1984; Pekár 2012). In the LME analyses, traits were treated as factors with fixed effects, and individuals and areoles were treated as factors with random effects. LMEs were fitted using the LME function in the NLME library of the R statistical program (R Development Core Team 2015). Results from the first step were used for other calculations. The influence of spine position on the plant body was analyzed using one-way nested ANOVA. Plant position was treated as a factor with fixed effects, and individuals and areoles were treated as factors with random effects. In the second step, minimal sample size (N) was calculated according to the following equation:

\[N = \dfrac{\sigma^{2}(t_{1-\alpha/2}(n-1))^{2}}{D^{2}}\]

where σ is the assumed standard deviation (SD) for the group, the (t_1-α/2_(n-1)) value is the quantile of the Student’s *t*-distribution for n-1 degrees of freedom and D is the desired margin of error. The interval limits for minimum sample size were taken as 10, 15 and 20% differences of the mean. Only spines sampled from the middle part of plant body were used for minimum sample size calculation. Calculations were performed in the R software environment (R Development Core Team 2015) and STATISTICA v. 10 (StatSoft, USA).

All acronyms, abbreviations and symbols are defined in Table 1.

**Table 1.** Spine and fiber traits, their abbreviations and units, examined throughout the study.

Trait	Explanation	Unit
Spines		
S_l_	Spine length	mm
S_w_	Spine width	mm
S_th_	Spine thickness	mm
S_a_	Spine cross-section area	mm^2^
S_c_	Spine circumference	mm
S_r_	Spine roundness	-
Fibers		
F_max_	Fiber maximum diameter	µm
F_min_	Fiber minimum diameter	µm
CW_th_	Cell wall thickness	µm
F_r_	Fiber roundness	-

## Results

### Spine surface structure in SEM

**Figure 2.**
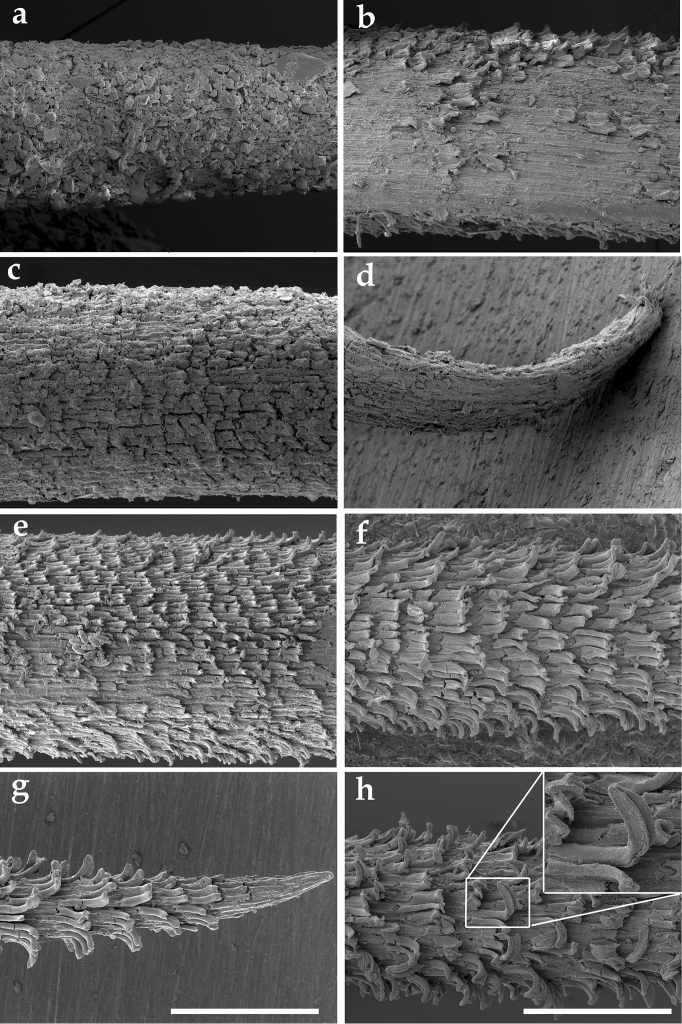
SEM images of upper, middle and basal part of Gymnocalycium kieslingii subsp. castaneum. **a** surface of spine from basal part covered with mineral deposition **b, c** surface of spine from basal part with a few epidermal cells **d** damaged, bent and deformed tip of spine from basal part **e** surface of spine from middle part with clearly visible epidermal cells sharply bent upward in upper part **f** surface of spine from upper part with very clearly visible and undamaged epidermal cells **g** undamaged top part of spine from upper part **h** epidermal cells of spine from upper part, bent lengthwise in its central part. Scale bar = 200 µm.

Spines in the basal part of the plant body were either completely covered with mineral deposition so that the surface structure was not recognizable or the surface was only partially visible (Fig. 2a, b). In rare cases, epidermal cells were still obvious (Fig. 2c). Spine tips were usually damaged, bent and deformed (Fig. 2d).

In the middle part of the plant body, the spine surface was only slightly damaged. The epidermal cells were clearly visible (Fig. 2e) and were only missing in a few cases. They were generally rectangular in shape (major to minor axis diameter ratio 2:1). Epidermal cells were usually sharply bent upward in the upper part (Fig. 2e) and arranged in regular transverse rows with different directions. In rare cases, deep fissures were observed.

Epidermal cells of spines in the upper part of plant body were very clearly visible and undamaged (Fig. 2f, g). Epidermal cells were irregular in both shape and size. They were flat on the spine tips and started to bend slightly to sharply at some distance from the spine tips (Fig. 2g). In some cases, they were also bent lengthwise in their central part (Fig. 2h). They were usually arranged in regular rows. The rows were wavy in shape and ran slightly upwards. No fissures were observed.

### Spine variation between individuals

Spine length (S_l_) ranged from 3 to 16 mm and spine cross-section area (S_a_) from 0.21 to 2.81 mm^2^ (Table 2). S_l_ and S_a_ had the largest coefficient of variation (CV) between individuals (Table 3). In contrast, spine roundness (S_r_) had the lowest CV (Table 3). In most cases, the shape was near-circular, as the mean S_r_ was 1.25 (Table 2). The mean fiber maximum diameter (F_max_) and fiber minimum diameter (F_min_) were 13.7 and 10.03 µm, respectively (Table 2). The studied fiber traits were less variable than spine traits between individuals (Table 3). Fiber roundness (F_r_) was the least variable trait (Table 3). The mean F_r_ value was 1.38 (Table 2), indicating fibers with a slightly ellipsoid shape. On the other hand, cell wall thickness (CW_th_) had the highest CV of the fiber traits (Table 3), ranging from 1.5 to 6.6 µm (Table 2).

**Table 2.** Mean (±SD), minimum and maximum values for all sampled spines (n=245), and mean values (±SD) for individuals (n = 15) and areoles (n = 45) of Gymnocalycium kieslingii subsp. castaneum. See Table 1 for explanation of spine and fiber traits.

Trait (unit)	mean±SD	min	max	mean±SD	mean±SD
All spines				Individuals	Areoles
Spines					
S_l_ (mm)	8.7±1.6	3	16	8.7±6.4	8.7±4.5
S_w_ (mm)	1.05±0.14	0.58	2.21	1.05±0.5	1.05±0.4
S_th_ (mm)	0.85±0.10	0.43	1.63	0.85±0.4	0.85±0.3
S_a_ (mm^2^)	0.73±0.16	0.21	2.81	0.72±0.6	0.73±0.5
S_c_ (mm)	3.05±0.37	1.69	6.16	3.05±1.4	3.07±1.1
S_r_	1.25±0.17	1.01	2.08	1.25±0.3	1.25±0.2
Fibers					
F_max_ (µm)	13.72±2.6	8.1	20.8	13.7±5.5	13.8±3.9
F_min_ (µm)	10.05±1.8	5.9	15.9	10.03±3.9	10.08±2.8
CW_th_ (µm)	3.82±0.99	1.5	6.6	3.8±2.2	3.8±1.5
F_r_	1.38±0.14	1.1	2.3	1.38±0.15	1.38±0.15

**Table 3.** Coefficient of variation (CV) for spine and fiber traits between all sampled spines, individuals and areoles. Values of CV above 40% are shown in bold. See Table 1 for explanation of spine and fiber traits.

Trait	CV (%)		
(unit)	All spines	Individuals	Areoles
	Spines		
S_l_ (mm)	18	**74**	**52**
S_w_ (mm)	13	**48**	38
S_th_ (mm)	12	**47**	35
S_a_ (mm^2^)	22	**83**	**68**
S_c_ (mm)	12	**46**	36
S_r_	10	24	16
Fibers			
F_max_ (µm)	19	40	28
F_min_ (µm)	18	39	28
CW_th_ (µm)	26	**58**	39
F_r_	10	11	11

### Spine variation between areoles and within the plant body

The fiber traits were less variable than spine traits between areoles (Table 3). Although the CV of spine and fiber traits between areoles was lower than that between individuals, it was higher than 30% in most cases (Table 3). The most and the least variable spine and fiber traits between areoles were the same as between individuals. The CV of S_a_ and S_l_ was the highest, whereas the CV for S_r_ and F_r_ was the lowest (Table 3). Spine and fiber traits were similar in the base and middle part of plant body. However, the upper part was significantly different from the other parts for almost all studied spine and fiber traits (except S_l_, S_r_ an F_r_) (Table 4). In general, studied traits were by 8–17% higher in the upper part than in the lower parts (Table 4).

**Table 4.** Influence of areole position on the plant body on spine and fiber traits. Bold letters show statistically significant differences between upper and basal part. See Table 1 for explanation of spine and fiber traits.

Trait (unit)	mean±SE			P-value
	basal part	middle part	upper part	
Spine				
S_l_ (mm)	8.3±0.28	8.8±0.21	9.2±0.29	0.12
S_w_ (mm)	1.02±0.02	1.03±0.02	1.11±0.03	**0.01**
S_th_ (mm)	0.82±0.01	0.84±0.02	0.89±0.03	**0.03**
S_a_ (mm^2^)	0.68±0.03	0.70±0.03	0.81±0.05	**0.01**
S_c_ (mm)	2.98±0.06	3.00±0.06	3.28±0.09	**0.01**
S_r_	1.26±0.02	1.22±0.01	1.21±0.02	0.45
Fiber				
F_max_ (µm)	13.0±0.3	13.6±0.4	14.7±0.3	**0.01**
F_min_ (µm)	9.5±0.3	10.06±0.4	10.7±0.6	**0.01**
CW_th_ (µm)	3.5±0.3	3.87±0.2	4.20±0.3	**0.01**
F_r_	1.4±0.04	1.37±0.04	1.4±0.11	0.38

### Spine variation within areoles

The CV of spine traits within an areole was lower than between areoles and individuals (Table 3 and Suppl. material 1). Nevertheless, a CV value higher than 15% was observed for S_a_ (91% of sampled areoles) and for S_l_ (60% of sampled areoles) (Suppl. material 1). The most and the least variable spine traits within an areole were almost the same as those between the areoles. On the other hand, the variation in fiber traits within an areole differed from the variation of fiber traits between the areoles. Contrary to the variation between areoles, F_r_ was the most and CW_th_ the least variable trait within an areole. A CV value higher than 15% was found in most cases for F_r_ (62% of sampled areoles) and for F_max_ (44% of sampled areoles). On the other hand, a CV of CW_th_ higher than 15% was only found for 7% of the sampled areoles.

**Figure 3.**
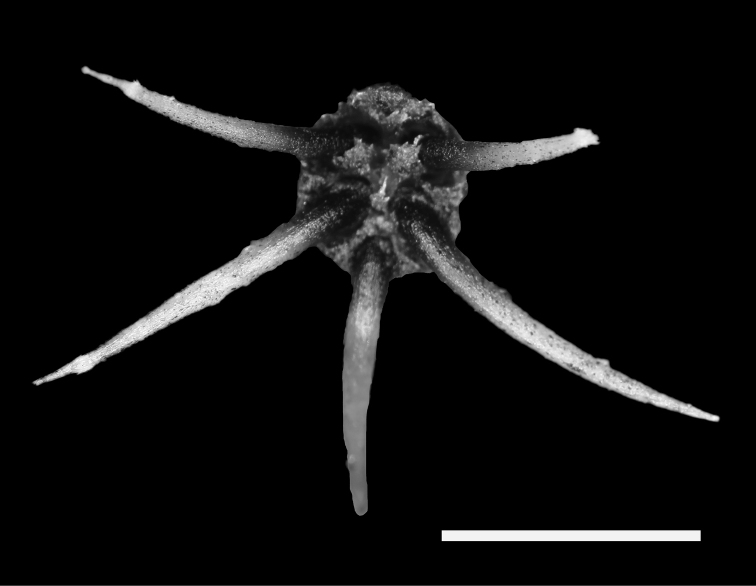
Typical radial arrangement of spines within an areole of Gymnocalycium kieslingii subsp. castaneum. All spines are straight or slightly curved to the body. The lowest spine is pointing downward. Scale bar = 5 mm.

Spines within an areole were distributed only marginally with radial arrangement (Fig. 3). All spines were straight or slightly curved to the body. The lower one pointing downward The number of spines per areole range from 3 to 7 spines and the most frequent number of spine per areole was 5 (51% from all areoles) (Suppl. material 1). Only 11% from all areoles had 3 and 4 spines.

### Minimum sample size

In general, calculated minimal sample sizes corresponded with the CVs of the studied traits. To study spine traits with 10% differences in the mean value, we should measure at least 52 spines (Table 5). However, if we studied only S_a_ or S_c_, 25 spines would be needed and even fewer spines would be needed for S_r_ (Table 5). In the case of fiber traits, the most variable trait was CW_th_, for which 70 spines should be used to obtain results within 10% differences in the mean value (Table 5). To study fiber diameter, 43 spines should be used and no more than 6 spines are needed for F_r_. The minimal sample size for the studied parameters would be on average 3.7 times lower, if 20% differences in the mean value were used (Table 5).

**Table 5.** Minimum sample size required to study specific spine and fiber traits. The interval limits were taken as 10, 15 and 20% differences of the mean.

Trait (unit)	sample size		
	mean ±10%	mean ±15%	mean ±20%
Spines			
S_l_ (mm)	52	23	13
S_w_ (mm)	40	18	10
S_th_ (mm)	34	15	9
S_a_ (mm^2^)	23	11	6
S_c_ (mm)	25	11	7
S_r_	1	1	1
Fibers			
F_max_ (µm)	43	19	11
F_min_ (µm)	40	18	10
CW_th_ (µm)	70	31	18
F_r_	6	2	2

## Discussion

### Spine surface

In the present study, the spine epidermal cells were usually bent upward, but flat shapes were also observed. Epidermal cells were usually arranged in regular transverse rows. The spine epidermis and mesophyll of several cacti have deep fissures, created during normal development (Mauseth 2006). These fissures are believed to play an important role in water absorption, but it has not yet been investigated what effect this process may have on the overall water balance of the plants (Mosco 2009). In Gymnocalycium kieslingii subsp. castaneum such fissures had only 9% of observed spines. Trichomes observed in other cactus species (Mauseth 2006, Řepka and Gebauer 2012) were also absent in Gymnocalycium kieslingii subsp. castaneum. It has been reported that differences in surface structure are related to age: young spines (situated in the upper part of the plant body) of Mammillaria scrippsiana var. armeria and *Echinopsis* sp. have a smooth surface, whereas older spines (situated in the lower parts of plant body) show a broken surface. We did not observe such differences, which may be specific characteristics of a particular genus of cacti. However, there were differences in spine surface damage and visibility. The spine surface was more visible and less damaged in spines from the upper part than in the lower parts. In the basal part, the spine surface was almost completely covered with mineral deposition (Fig. 2a). Thus, spines situated in the upper part of plant bodies are the most appropriate for surface analysis and taxonomic use.

### Spine variation between individuals and areoles

Spines develop from lateral buds (areoles) and vary considerably across species in number, length, width and thickness (Mauseth 2006, Mosco 2009). This is an extreme form of leaf modification (Mauseth 2006). The number of spines per areole of Gymnocalycium kieslingii subsp. castaneum is described as ranging from 5 to 7 (Ferrari 1980). In the present study, areoles with 3 and 4 spines were also found in 11% from all areoles. The spines shape was the least variable trait and it was mostly circular. This is in accordance with Mauseth (2006), who mentioned that cactus spines are frequently circular in cross-section. On the other hand, S_l_ and S_a_ were the most variable spine traits. In another study, spine length was also found to be the most variable trait in 30 *Aylostera* and Rebutia (Cactoideae) hybrids (Mihalte and Sestras 2012). It has already been found that spine variation occurs even within a single species, reflecting environmental conditions during cactus growth (e.g. Nobel 1988, English et al. 2007, Menezes et al. 2015). For example, S_l_ was found to be positively correlated with rainfall (Menezes et al. 2015). It is obvious that longer and thicker spines will require more photosynthates for its development. On the other hand, such a spine increment would reduce the interception of photosynthetic active radiation, which would then reduce photosynthetic productivity (Nobel 1983). However, abundant spines shade the photosynthetic cortex from intense insolation and UV radiation to avoid high-temperature extremes (Loik 2008). Thus, the large variation of S_l_ and S_a_ found in our study may be partly explained by a sensitive plant regulation system that balances between positive and negative spine functions. However, the factors controlling morphogenesis in the basal meristems of spines are still unknown (Mauseth 2006) and the identification of genes and their expression will be an important step towards our understanding of the spine development.

Two main fiber shapes in spine cross-sections (i.e. folded and pillar) were described by Schlegel (2009). Fibers of Gymnocalycium kieslingii subsp. castaneum were mostly oval and had a pillar structure. In our study, the mean fiber maximum and minimum diameters were in the range reported for *Echinocactus grusonii* (Huang and Guo 2013), *Opuntia ficus-indica* (Vignon et al. 2004) and *Turbinicarpus* sp. div. (Mosco 2009), which had spines composed of fibers with diameters of 5–15, 6–10 and 6–18 µm, respectively. There are only two studies including measurements of cell wall thickness of fibers (CW_th_). *Turbinicarpus* species had a mean CW_th_ of 0.7 to 5.9 µm (Mosco 2009), and *Escobaria* species had a mean CW_th_ of 3.3–4.0 µm (Řepka and Gebauer 2012). These values are slightly lower than for Gymnocalycium kieslingii subsp. castaneum.

### Spine variation within areoles

Although a large variation of spine traits within single species has been described (Peharec et al. 2010), there is no extant report describing spine variation within an areole. Since the spines grow from the same lateral bud, low variation of anatomical and morphological parameters would be expected. This hypothesis was only partly supported in our study, as the spine and fiber variation within areoles was lower than between areoles, but the CV for different studied traits within an areole was still high. For example, the CV for S_a_ was even higher than 40% in a few cases (Suppl. material 1). Thus, it seems that even within a single areole, the function of the basal meristem of the spine is very sensitive to environmental and internal stimuli.

### Spine variation on the plant body

The areole position on the plant body could be related to age, since new spines develop on top of the cactus body, whereas the oldest spines are situated in the basal part. Thus, variation in spine traits from different positions on the plant body can be expected due to different environmental conditions during spine development. For example the stable isotope composition of spines produced serially from the apex of the long-lived columnar species *Carnegiea gigantea* revealed the past physiological and climatic variation (English et al. 2007). This corresponds with our results, as almost all spine and fiber traits (except for S_l_, S_r_ and F_r_) had different values in the lower parts than in the upper part of the plant body. Nevertheless, the growth of cacti is very slow, and determination of their age without direct observation is difficult (Martinez del Rio et al. 1995).

### Minimum sample size

Although spine variation is known even in single species, spine sample sizes used by different authors are very variable. For example, anatomical studies use 2 spines (Mosco 2009), morphological studies use 17 spines (Peharec et al. 2010), and mechanical studies usually use 2–22 spines (Schlegel 2009, Huang and Guo 2013). In our study, we found that the minimum sample size was largely influenced by the studied spine and fiber traits, and ranged from 1 to 70 spines, if 10% difference in the mean value was taken in account. Thus, for spine sample collection, the spine/fiber trait to be studied is crucial. In our case, fewer spines (taken from the middle part of the cactus body) would be needed to describe S_r_ (1 spine), F_r_ (6 spines), S_a_ (23 spines) and S_c_ (25 spines). In contrast, more spines should be collected to study CW_th_ (70 spines), S_l_ (52 spines), F_max_ (43 spines) and S_w_ (40 spines). These sample sizes may, however, only apply to spines collected from different plants within a single population. We should note that more similar studies on other cactus species or on the same species, but growing in different environment conditions are needed to make generalizations for spine sample collection in the field. This is important task for the correct delimitation and identification of cactus species especially if cultivated specimens were used as sources of evidence in taxonomy (e.g. Schmalzel et al. 2004).

## Conclusion

Our study of 15 cactus individuals, 45 areoles and 245 spines showed that spine and fiber traits are highly variable. The areole position on the plant body was an important factor in most of the studied spine and fiber traits (Question 1). The spine and fiber variation within an areole was lower than between areoles, but the variation was still high (Question 2). The minimum sample size was largely influenced by the examined spine and fiber trait, ranging from 1 to 70 spines (mean ± 10%) (Question 3). The large spine and fiber variation between individuals and even within single areoles observed in this study indicates a very complex spine morphogenesis. We encourage a further research focus on the spine and fiber variation in other cacti species, but also on the factors controlling the basal meristem function and gene expression in spines.

## Supplementary materials

Supplementary material 1 Table S1. Coefficient of variation (CV) of spine and fiber traits within areoles. Data type: specimens data Explanation note: The number of spines within an areole is given in brackets (n). Depicted are CV values above 15%. See Table 1 for explanation of spine and fiber traits. This dataset is made available under the Open Database License (http://opendatacommons.org/licenses/odbl/1.0/). The Open Database License (ODbL) is a license agreement intended to allow users to freely share, modify, and use this Dataset while maintaining this same freedom for others, provided that the original source and author(s) are credited.Roman Gebauer, Radomír Řepka, Radek Šmudla, Miroslava Mamoňová, Jaroslav Ďurkovič
