# Supplementary material for: Anatomical and morphological spine variation in Gymnocalycium kieslingii subsp. castaneum (Cactaceae)
Source: PhytoKeys. 2016 Aug 18;(69):1–15. doi: 10.3897/phytokeys.69.8847 (PMC5029135; doi:10.3897/phytokeys.69.8847)
Supplement: Supplementary material 1 — Table S1. Coefficient of variation (CV) of spine and fiber traits within areoles. [file phytokeys-069-001-s001.docx]

Table S1. Coefficient of variation (CV) of spine and fiber traits within areoles. The number of spines within an areole is given in brackets (n). Depicted are CV values above 15%. See Table 1 for explanation of spine and fiber traits.

| Areola (n) | S_l_ | S_w_ | S_th_ | S_a_ | S_c_ | S_r_ | F_max_ | F_min_ | CW_th_ | F_r_ |
| --- | --- | --- | --- | --- | --- | --- | --- | --- | --- | --- |
|  |  | CV (%) | | | | | | | | |
| 1(7) | 10 | 6 | **17** | **22** | 10 | 14 | **24** | **28** | 7 | **31** |
| (7) | **19** | 11 | 14 | **22** | 11 | 11 | 7 | 14 | 9 | **24** |
| 3(6) | **19** | **22** | **19** | **44** | **21** | 9 | 6 | 4 | 10 | 4 |
| 4(3) | 14 | 3 | 3 | 11 | 6 | 1 | **21** | 14 | **18** | **28** |
| 5(5) | **30** | **22** | **25** | **45** | **23** | 6 | **18** | 14 | 9 | **20** |
| 6(5) | **28** | **23** | **22** | **45** | **23** | 8 | 2 | 8 | 6 | 11 |
| 7(5) | **20** | **15** | 11 | **24** | **16** | 6 | **19** | **20** | 7 | **24** |
| 8(7) | **16** | 15 | **17** | **30** | 14 | 13 | **17** | 12 | 15 | **18** |
| 9(6) | **18** | **16** | **18** | **30** | **16** | 13 | 11 | 11 | 2 | 8 |
| 10(5) | 5 | 6 | **21** | **18** | 9 | **28** | 5 | **21** | **30** | **30** |
| 11(7) | 6 | 12 | 9 | **17** | 7 | **16** | 4 | 6 | 2 | 8 |
| 12(5) | 14 | **17** | 13 | **18** | 11 | **18** | 3 | 12 | 11 | **18** |
| 13(6) | 15 | **19** | **16** | **31** | **17** | 14 | **16** | **16** | 7 | 11 |
| 14(7) | **17** | **16** | 15 | **30** | 14 | 13 | **16** | **17** | 5 | **18** |
| 15(5) | 14 | 5 | 7 | 10 | 6 | 7 | **23** | 9 | **22** | **17** |
| 16(4) | 10 | 6 | 11 | **17** | 8 | 8 | 11 | 13 | 3 | **16** |
| 17(7) | **20** | 11 | 9 | **18** | 9 | 9 | **21** | 14 | 9 | 11 |
| 18(7) | **25** | **16** | **16** | **32** | 15 | 9 | 11 | 8 | 6 | 11 |
| 19(5) | 12 | **19** | **18** | **37** | **19** | 3 | **16** | **17** | 7 | **16** |
| 20(4) | **23** | 13 | **19** | **29** | 15 | 11 | **17** | 14 | 6 | **16** |
| 21(6) | **28** | 11 | **16** | **25** | 12 | 14 | 12 | 11 | 3 | 14 |
| 22(5) | **26** | 7 | **22** | **26** | 12 | **18** | **20** | 11 | 12 | **18** |
| 23(5) | 11 | 12 | 7 | **17** | 9 | 8 | **17** | 10 | 3 | 15 |
| 24(6) | **18** | 9 | 11 | **19** | 9 | 6 | 12 | 14 | 5 | **16** |
| 25(3) | **16** | **35** | **28** | **64** | **32** | 14 | **16** | 14 | 6 | **20** |
| 26(7) | **16** | 9 | 13 | 17 | 8 | **17** | 11 | 6 | 10 | 11 |
| 27(7) | 9 | 14 | 13 | **24** | 12 | 11 | **19** | **20** | 4 | **28** |
| 28(5) | 13 | 12 | 11 | **22** | 11 | 4 | **24** | **23** | 3 | **28** |
| 29(7) | **20** | **16** | **24** | **39** | **19** | 11 | 9 | 15 | 8 | **22** |
| 30(6) | **18** | **18** | **17** | **33** | **17** | 7 | 10 | 13 | 4 | **21** |
| 31(5) | **20** | **16** | **28** | **44** | **19** | **22** | **16** | 15 | 10 | **30** |
| 32(5) | **20** | **18** | 12 | **30** | 14 | 9 | 14 | 9 | 7 | 9 |
| 33(5) | **26** | **20** | **23** | **40** | **19** | **22** | 11 | 11 | 9 | 10 |
| 34(5) | **19** | 9 | **17** | **28** | 12 | 7 | 12 | 13 | 8 | **28** |
| 35(5) | **18** | 13 | 11 | **22** | 12 | 5 | 7 | 7 | 9 | 9 |
| 36(7) | **17** | **30** | **34** | **57** | **32** | 10 | 13 | **17** | 5 | **37** |
| 37(5) | 7 | 15 | 14 | **29** | 14 | 3 | 8 | 9 | 4 | 5 |
| 38(5) | **23** | **19** | **16** | **31** | **17** | 9 | 10 | 13 | 3 | 12 |
| 39(5) | **25** | 15 | 15 | **30** | 15 | 7 | 15 | 14 | 5 | **17** |
| 40(3) | 10 | 4 | 0 | 4 | 3 | 5 | 14 | 10 | 5 | **18** |
| 41(5) | 7 | 15 | **22** | **35** | **17** | 12 | **16** | 8 | 9 | 13 |
| 42(5) | 10 | 7 | **19** | **25** | 11 | **17** | 2 | 3 | 1 | 1 |
| 43(5) | 12 | 15 | 13 | **26** | 14 | 8 | **17** | **17** | 5 | **22** |
| 44(5) | **19** | 7 | 13 | **19** | 8 | 8 | **23** | **28** | 7 | **30** |
| 45(5) | 9 | 7 | 8 | 15 | 7 | 3 | 15 | **17** | 4 | **29** |
